# Intraoral Adult Rhabdomyoma: A Case Report

**DOI:** 10.1155/2013/741548

**Published:** 2013-10-30

**Authors:** Ana Amelia Souza, Vera Cavalcanti de Araújo, Fabricio Passador Santos, Elizabeth Ferreira Martinez, Jose Ferreira de Menezes Filho, Ney Soares de Araujo, Andresa Borges Soares

**Affiliations:** ^1^Department of Oral Pathology, São Leopoldo Mandic Institute and Research Center, Rua José Rocha Junqueira 13 Ponte Preta, 13045-755 Campinas, SP, Brazil; ^2^Instituto Tocatinense Presidente Antonio Carlos-Araguaína, Avenida Filadélfia 568, 77816-540 Araguaína, TO, Brazil

## Abstract

A case of adult rhabdomyoma is reported. The lesion is a rare benign tumor of skeletal muscle origin which occurs predominantly in the head and neck region. In the present case, the clinical diagnosis favored a benign salivary gland tumor. Histologically, the tumor was composed of large round, oval, and polygonal cells of varying size with abundant pale, eosinophilic, fine, granular cytoplasm with peripherally located nuclei. Immunohistochemically, the lesion was positive for muscle-specific actin, smooth muscle actin, desmin, S100 protein, and Masson's trichrome. Electron microscopic examination confirmed the presence of numerous myofibrils. The lesion was treated by surgical resection. The clinical, histological, immunohistochemical, and ultrastructural features are discussed in this study.

## 1. Introduction

Rhabdomyomas are rare benign tumors derived from striated muscle, and can be classified generally into two types: cardiac type and extracardiac type. Cardiac rhabdomyoma occurs almost exclusively in the pediatric age group and may be associated with tuberous sclerosis, neurofibromatosis, and sebaceous adenomas. Current opinion is that the cardiac type represents a hamartomatous growth [1].

Extracardiac rhabdomyoma can be divided into three groups (adult, fetal, and genital types) with regards to clinical and morphological differences. The adult type is characterized by a slowly growing mass typically seen in the head and neck of elderly patients. In the fetal type, the lesion also typically involves the head and neck region and tends to occur at younger ages. The genital type is almost always found in the vulvovaginal region of middle-aged women [2]. The fetal and vaginal types are morphologically similar [3]. Seventy-seven percent of all extracardiac rhabdomyomas occur in the head and neck and 14% in the genital region [4].

 The adult rhabdomyoma is the most common form of extracardiac rhabdomyoma. The tumor arises mainly within the head and neck region where it is believed to originate from the skeletal musculature of the third and fourth branchial arches. It is so called because of the histological resemblance to mature skeletal muscle cells [5]. These tumors are usually solitary; however, several authors have reported cases of adult multifocal extracardiac rhabdomyomas [6, 7]. In this paper we report a rare case of an adult rhabdomyoma originating in the floor of the mouth.

## 2. Case Report

The patient, a 40-year-old white woman, came to dentistry complaining of a painless swelling in the left floor of mouth that had been present for 6 months. The patient's medical history and extraoral examination did not show significant alteration.

**Figure 1:**
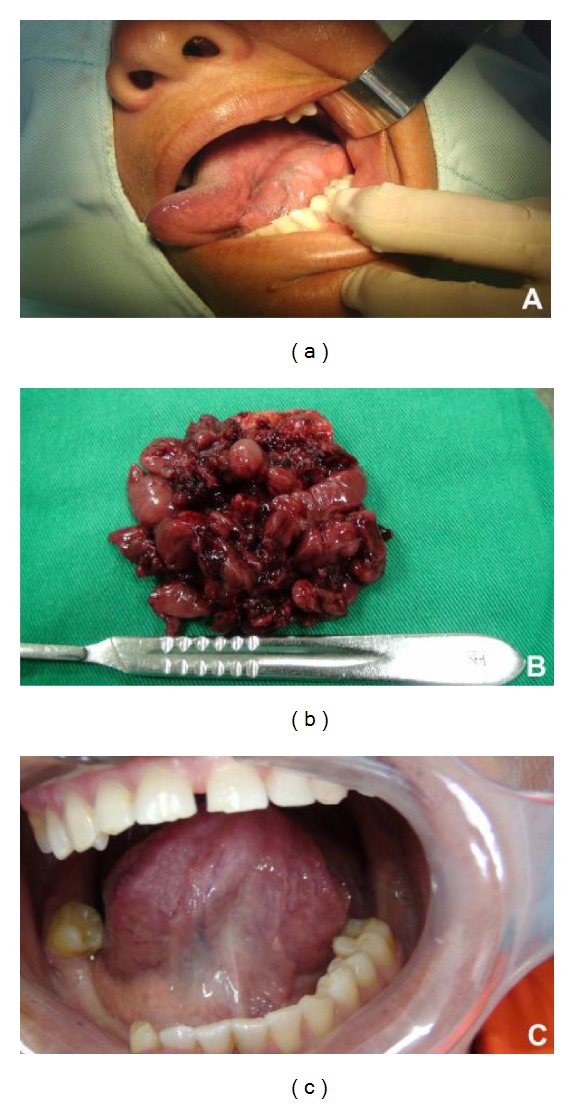
Clinical appearance of the lesion. Extensive soft tissue mass in the left floor of the mouth (a). Gross specimen revealed lobules of tumor tissue (b). Clinical aspect after surgery (c).

Intraoral examination revealed a soft, painless, nontender mass in the left floor of the mouth, with extension posteriorly into the supraglotic region (Figure 1(a)). A computerized tomography examination confirmed the existence of hyperdense lesion. The lesion appeared as a single homogenous mass in the lower middle portion in the floor of the mouth. The clinical diagnosis was a benign salivary gland tumor and an incisional biopsy was performed.

**Figure 2:**
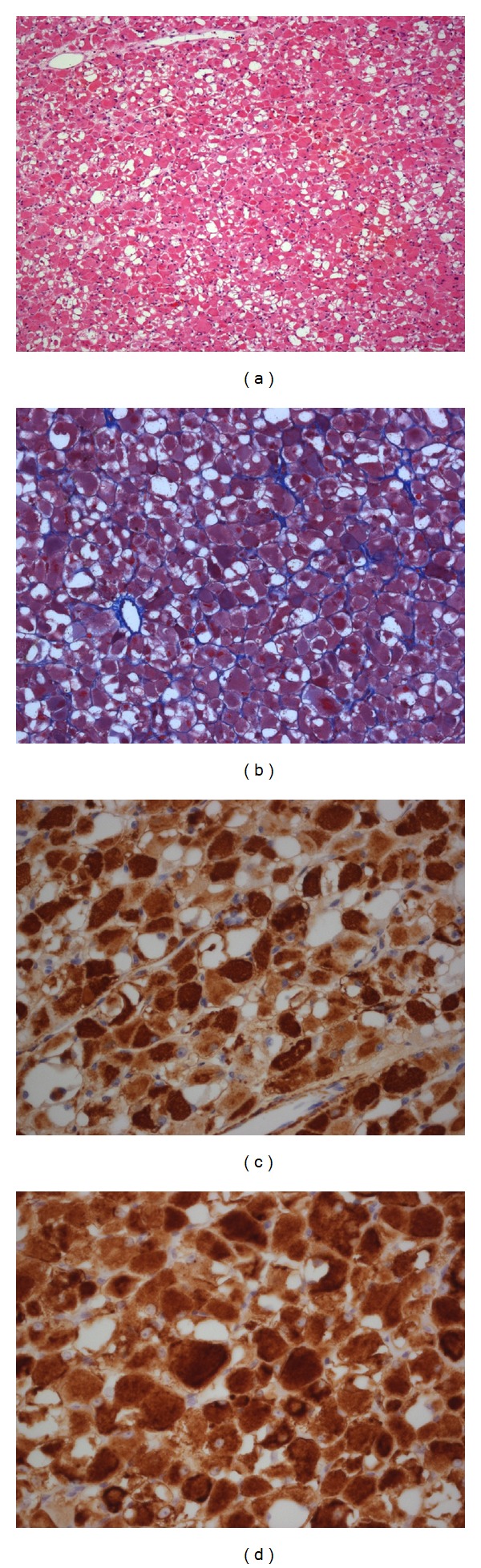
Histopathology and immunohistochemistry of the lesion. Microscopic examination showing large polygonal cells with eosinophilic and vacuolated cytoplasm (hematoxylin and eosin stain; original magnification: 20x) (a), Masson's trichrome staining demonstrating red round, oval, and polygonal cells (original magnification 20x) (b), immunohistochemical stain showing strong positivity against muscle-specific actin (c), and desmin antigens (d) (original magnification, 40x).

The specimen was fixed with 10% formalin, and paraffin sections were prepared for light microscopy using routine procedures. The initial sections were stained with hematoxylin and eosin (H&E). Microscopic examination revealed a tumor composed of large round, oval, and polygonal cells of varying sizes with abundant, pale, eosinophilic, fine, granular cytoplasm with peripherally located nuclei. Many tumor cells were vacuolated, with the extent of vacuolization varying from one cell to another. In most cells the vacuoles were located in the periphery, and in a few cells the extensive cytoplasmic vacuolization resulted in a spider web appearance. Mitoses and necrosis were absent. Masson's trichrome staining demonstrated red round, oval, and polygonal cells (Figures 2(a) and 2(b)).

Immunohistochemistry demonstrated tumor cells with strong reactivity to antibodies directed against muscle-specific actin (clone HHF35, Dako Cytomation) and desmin (clone D33, Dako Cytomation) as well as focal reactivity for smooth muscle actin (clone 1A4, Dako Cytomation) (Figures 2(c) and 2(d)).

**Figure 3:**
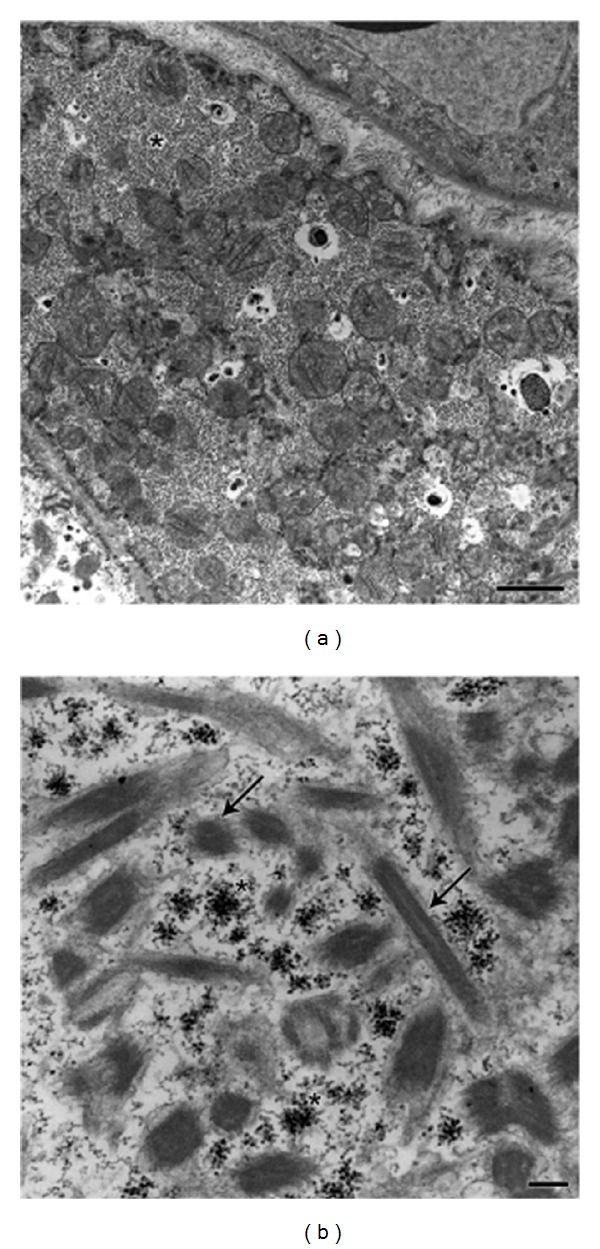
Transmission electron micrograph of rhabdomyoma. Numerous mitochondria and glycogen inclusions (asterisk) are seen (a) among the myofibrils (arrows) (b). Bars: A = 2,5 *µ*m; B = 1 *µ*m.

The ultrastructural morphological examination revealed numerous myofibrils displayed in a longitudinal and transversal pattern. In addition, some glycogen inclusions and mitochondria were identified (Figures 3(a) and 3(b)).

Based on these features, a final diagnosis adult rhabdomyoma was rendered and the treatment of choice was local excision.

The tumor was successfully removed en bloc under general anesthesia (Figure 1(b)). Surgical margins were tumor-free. No signs of local recurrence were observed two years after the surgical procedure (Figure 1(c)).

## 3. Discussion

The case reported fulfills the clinical, histological, immunohistochemical, and ultrastructural criteria for the diagnoses of adult rhabdomyoma, which is a rare benign tumor. The term rhabdomyoma was introduced by Zenker in 1864 [2, 5, 8–10].

In the present case, the lesion was presented in a 40 year-old woman, located in the floor of the mouth. The neoplasm has a predilection for the head and neck. The most commonly affected site is the neck and other sites include the oral cavity (base of the tongue and floor of mouth), pharynx, and larynx [6, 10]. A review of the literature suggests that these tumors are most frequently found in muscles derived from the pharyngeal arches [11].

The mean age of adult rhabdomyomas is 50 years (reported age range from 2 to 80 years); it occurs more commonly in males than females (ratio 4 : 1), and there is no predilection for any particular race [12]. 

The lesion usually presents as a smooth, movable, solitary (but occasionally multifocal), asymptomatic, round, or polypoid nodule in the head and neck region, or as a circumscribed intramuscular mass in the tongue, the sublingual region, lips, cheek, orbit, or submandibular region that is neither tender nor painful [13]. However, it may compress or displace the tongue or may protrude and partially obstruct the pharynx or larynx. As a consequence, it may cause hoarseness or progressive difficulty in breathing or swallowing. Multifocal rhabdomyomas may occur simultaneously, or the lesions may develop several years apart. Rhabdomyomas are slowly growing, and the lesions vary in size from a few millimeters to 15 cm [5, 14]. In our case, the patient presented with a painless solitary nodule; however, the nodule did make swallowing difficult for her. 

Tissue specimens from the current case showed histological features typical to adult rhabdomyoma. Histologically, the adult rhabdomyoma type is characterized by a sheet-like proliferation of tightly packed, large polygonal to round cells with abundant, deeply eosinophilic, granular cytoplasm with one or two peripherally placed vesicular nuclei; prominent nucleoli may occasionally be identified. Many cells show cytoplasmic vacuolization due to intracytoplasmic glycogen accumulation. Some cells have a small central acidophilic cytoplasmic mass connected by thin strands of cytoplasm to a condensed rim of cytoplasm at the periphery. These so-called “spider-cells” are more prevalent in cardiac rhabdomyomas than in extracardiac rhabdomyomas. Cross-striations are usually readily identifiable. Mitoses and necrosis are absent [1, 6, 15]. PAS staining with diastase digestion reveals large numbers of tumor cells rich in glycogen particles. This likely reflects the degree of maturation of the tumor cells because fetal rhabdomyomas are composed predominantly of immature skeletal muscle cells with little or no glycogen [5].

The results of our immunohistochemical studies are in agreement with previously published data that show positive immunoreactivity against desmin and muscle-specific actin. These findings are consistent with the known staining properties of muscle cells but are not specific for neoplasia. Desmin is present in muscle cells of all types but has a greater staining reliability for skeletal muscle. HHF35 is a specific and sensitive marker for tumors of muscle origin [16]. The adult rhabdomyoma showed variable immunoreactivity for vimentin, S100, and smooth muscle actin [4, 6]. 

Transmission electron microscope examination revealed features of rhabdomyoma. Thick and thin myofilaments were found in varying proportions, as well as degrees of organization disposable among numerous mitochondria, as well as glycogen inclusions. Although immunocytochemistry may provide evidence to support the pathologist's impression in many cases; electron microscopy is especially important in undifferentiated tumors when immunocytochemistry is noncontributory or when there is aberrant, confusing immunoreactivity with several different, unrelated antibodies [6, 17–22].

The main consideration of the histological differential diagnosis of this tumor includes lesions consisting of cells having abundant eosinophilic cytoplasm. One such entity is the granular cell tumor; both granular cell tumor and rhabdomyoma can form syncytial cell clusters with abundant eosinophilic cytoplasm. Immunohistochemistry can also be helpful in distinguishing the two lesions. Granular cell tumors show diffuse S-100 positivity and desmin negativity. Other entities that should be considered are salivary gland tumors, namely, acinic cell carcinoma or oncocytoma [2, 23]. Malignant tumors showing rhabdoid differentiation such as rhabdomyosarcoma and malignant rhabdoid tumor may also be included. Generally, the nuclear atypia and pleomorphism present allow for separation of these malignant tumors from rhabdomyoma [13]. 

The treatment of choice for rhabdomyoma is surgical excision. Recurrence occurred in 16% of cases reported in the literature, but most were caused by incomplete removal. Recurrence may develop after many years because the tumor grows extremely slowly. There is also the possibility that a recurrence actually represents a new lesion. Malignant transformation or locally aggressive behavior has yet to be reported with any of these lesions. Patients after resection require regular follow-up visits [5, 23–25].
